# Supplementary material for: Dynamic tuning of optical absorbers for accelerated solar-thermal energy storage
Source: Nat Commun. 2017 Nov 14;8:1478. doi: 10.1038/s41467-017-01618-w (PMC5684399; doi:10.1038/s41467-017-01618-w)
Supplement: Supplementary file 1 — Supplementary Information [file 41467_2017_1618_MOESM1_ESM.pdf]

Description of Supplementary

File Name: Supplementary Information

Description: Supplementary Figures, Supplementary Notes and Supplementary References

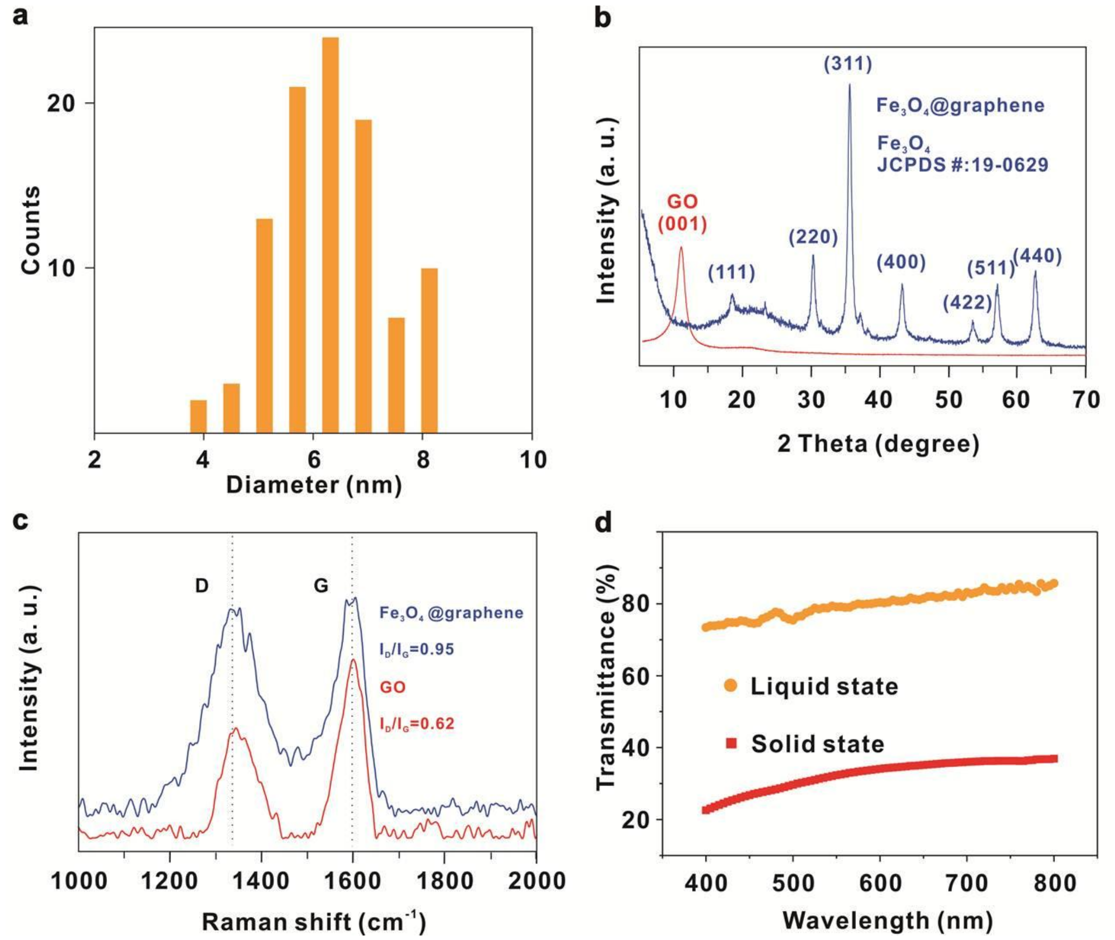

Supplementary Figure 1 | Characterization of Fe_3O_4@graphene hybrid particles and paraffin composite. **a**, Histogram of Fe_3O_4 particle size distribution on the surface of graphene. **b**, X-ray diffraction (XRD) spectra of graphene oxide (GO) and Fe_3O_4@graphene. **c**, Raman spectra of GO and Fe_3O_4@graphene. **d**, Transmittance spectra of paraffin composites (0.02 wt% Fe_3O_4@graphene) at melted liquid state and solid state.

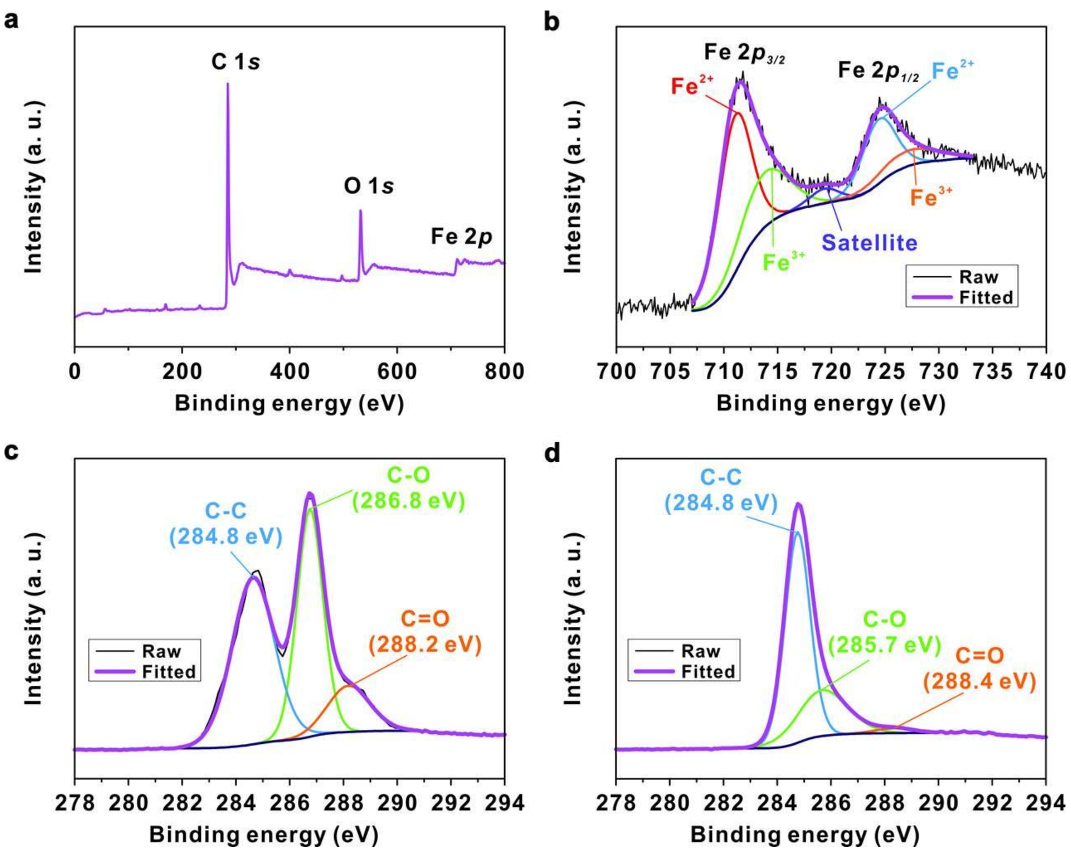

Supplementary Figure 2 | X-ray photoelectron spectroscopy (XPS) characterization of Fe_3O_4@graphene hybrid nanoparticles (NPs). a, XPS survey of hybrid NPs. **b**, XPS spectrum of Fe 2*p* level for hybrid NPs. The peaks at 711 eV and 724.4 eV correspond to the Fe^{2+} binding state while those at 713.8 eV and 727.1 eV are assigned to Fe^{3+} binding state in the Fe_3O_4 phase. The small peak at 719.3 eV is a satellite peak that might be assigned to Fe^{3+} in a minor γ-Fe_2O_3 phase. **c**, XPS spectrum of C 1*s* level for graphene oxide (GO). **d**, XPS spectrum of C 1*s* level for hybrid NPs. The peak area of C-O and C=O binding state was greatly decreased after conversion from GO to Fe_3O_4@graphene.

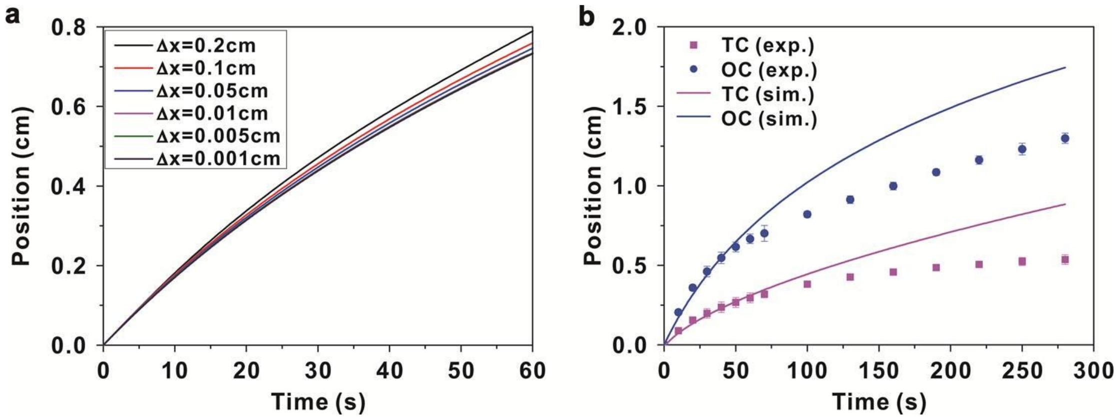

Supplementary Figure 3 | Theoretical modeling of charging process. a, Effect of the step size (Δx) on simulated solid/liquid charging interface movement for optical charging (OC) mode. When the step size approaches 0.01 cm the simulated movement of charging interface converges. **b,** Simulated and experimental movement of solid/liquid charging interface under thermal charging (TC) and OC mode.

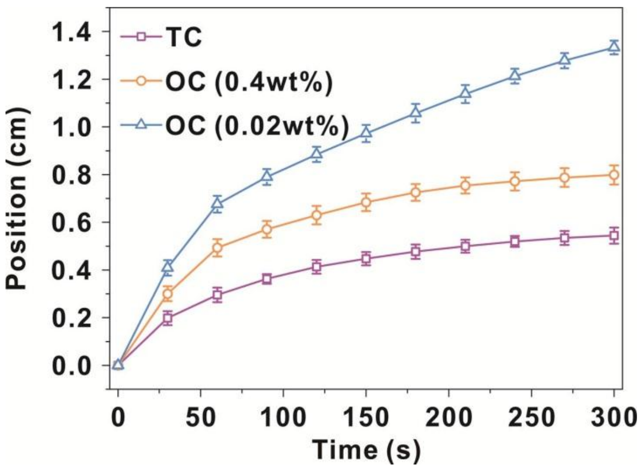

Supplementary Figure 4 | Advancement of charging interface of paraffin composite (0.4 wt% Fe_3O_4@graphene) under optical charging (OC) mode. The movement of charging interface for composite with 0.02 wt% of hybrid nanoparticles charged under OC mode (blue line) and paraffin composites charged under TC mode (purple line) was also provided for comparison.

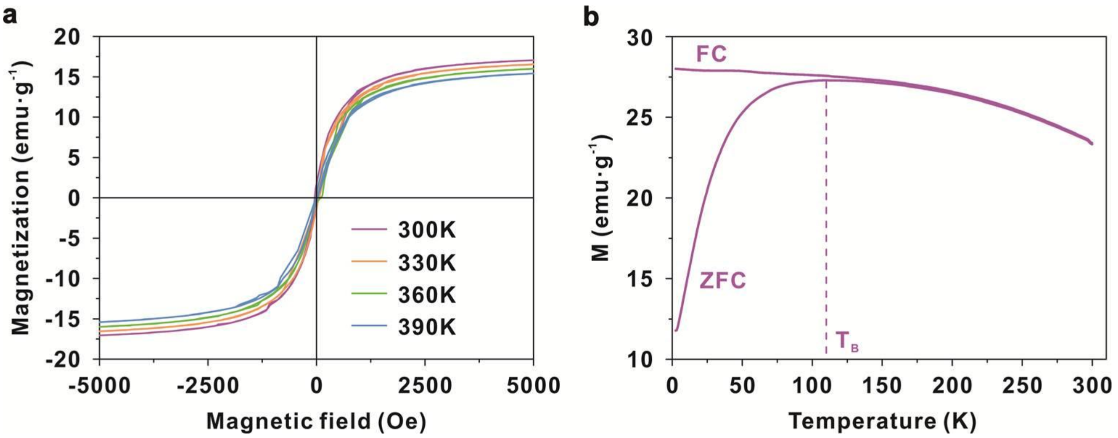

Supplementary Figure 5 | Magnetic characterization of Fe_3O_4@graphene nanoparticles. **a**, Magnified magnetization curves of Fe_3O_4@graphene nanoparticles. **b**, Field Cooling-Zero Field Cooling (FC-ZFC) curves of Fe_3O_4@graphene nanoparticle measured at 500 Oe showing a superparamagnetic blocking temperature (T_B) at 110 K.

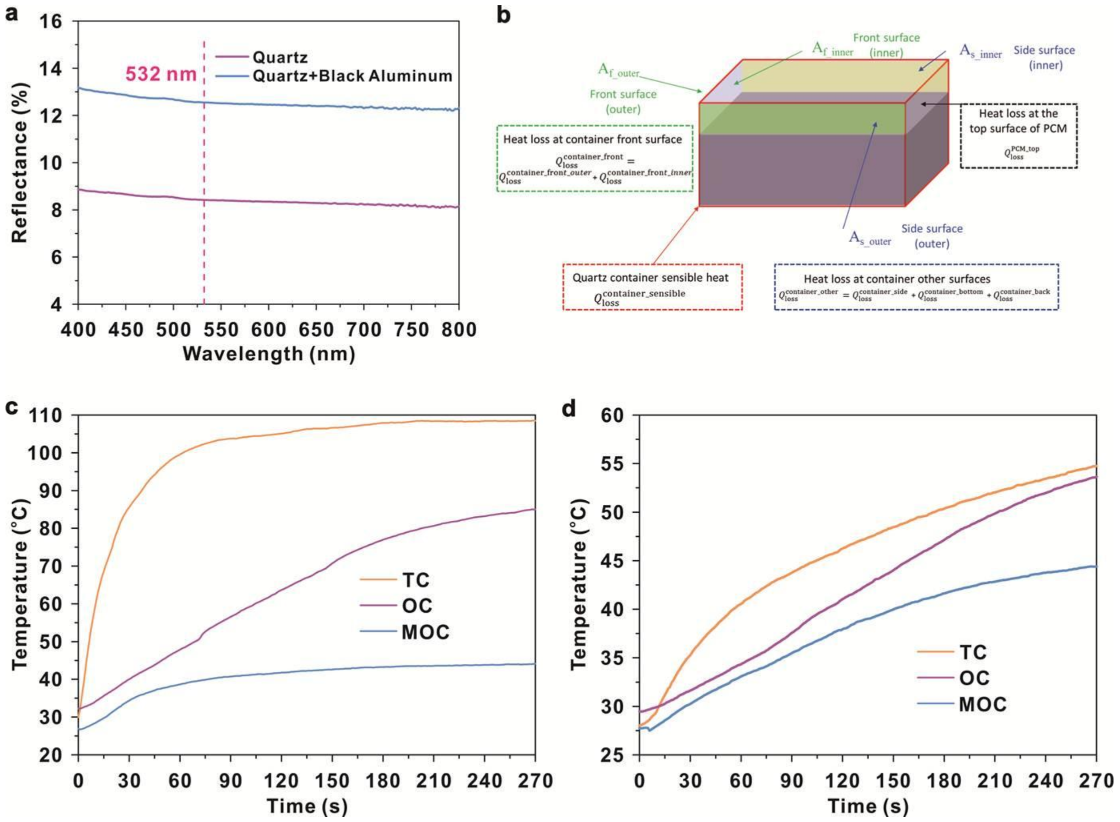

Supplementary Figure 6 | Heat loss analysis of paraffin composite system during the charging process. a, Reflection loss of incident laser light on quartz and quartz-black aluminum surfaces. **b**, Possible heat losses from the charging system. **c**, Average temperature of the front surface of container for three charging modes. **d**, Average temperature of the side surface of container for three charging modes.

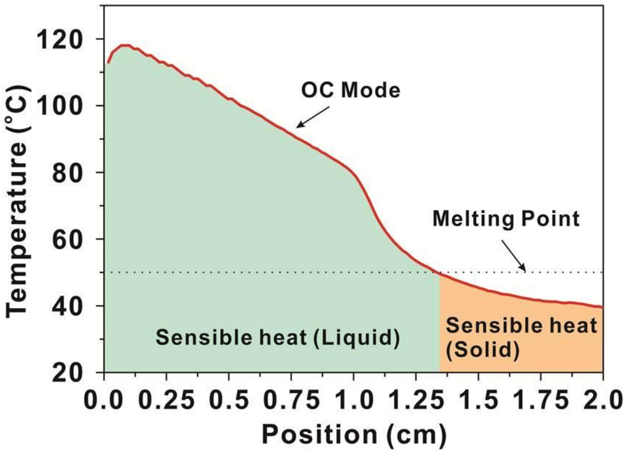

Supplementary Figure 7 | Scheme for calculation of stored sensible and latent heat in the charged paraffin composites. The green area marks melted paraffin region and the yellow area marks solid paraffin region.

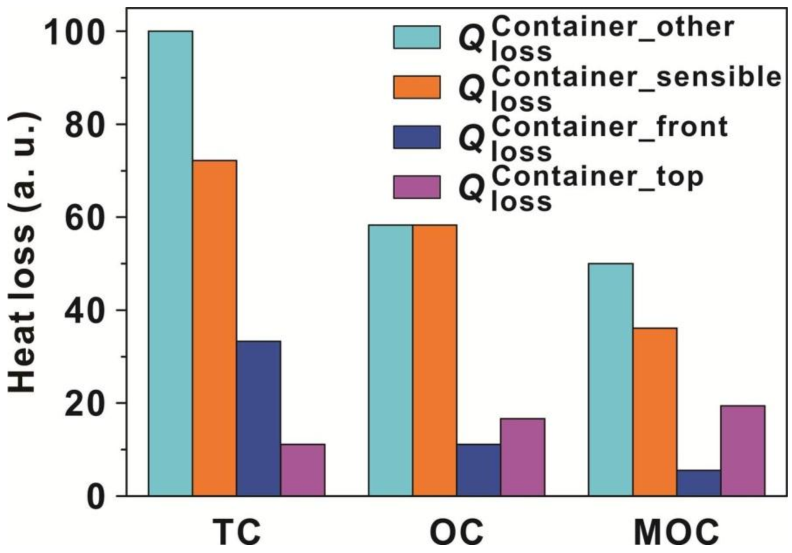

Supplementary Figure 8 | Estimation of heat losses in three different charging modes without thermal insulation.

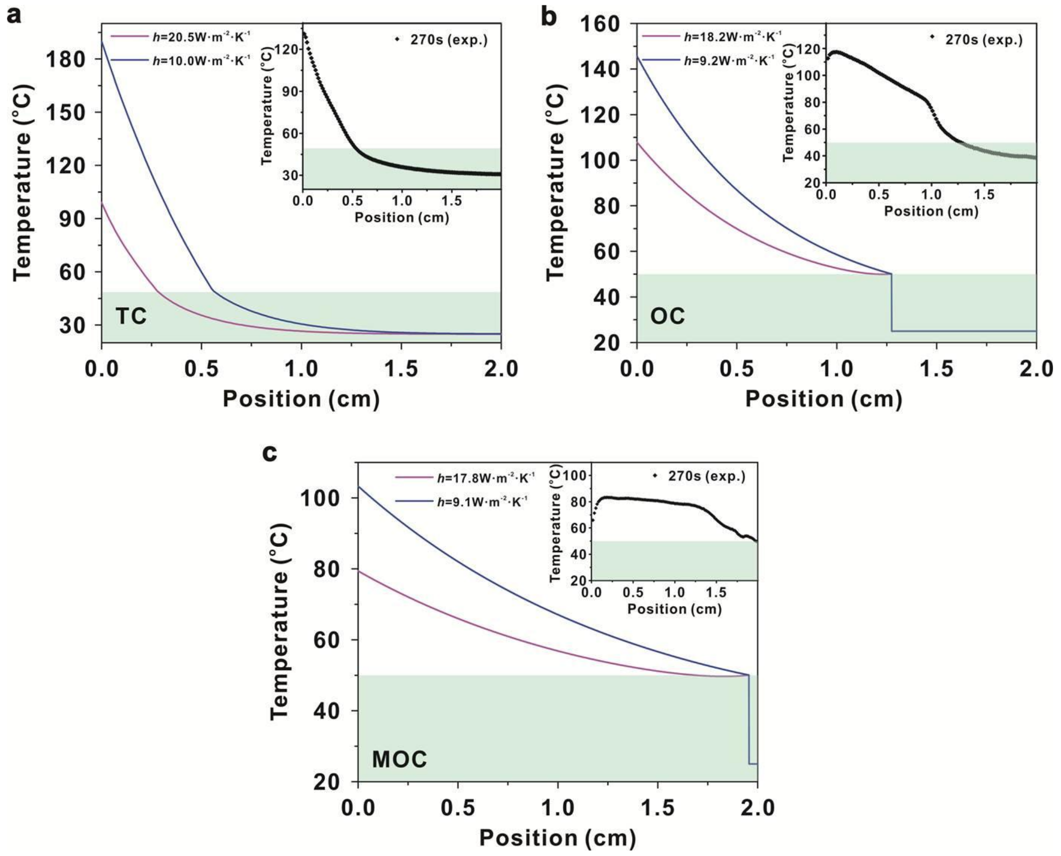

Supplementary Figure 9 | Calculated and measured temperature distribution profiles after charging for 270 s under different modes. a, Thermal charging (TC) mode. **b,** Optical charging (OC) mode. **c,** Magnetically-enhanced optical charging (MOC) mode.

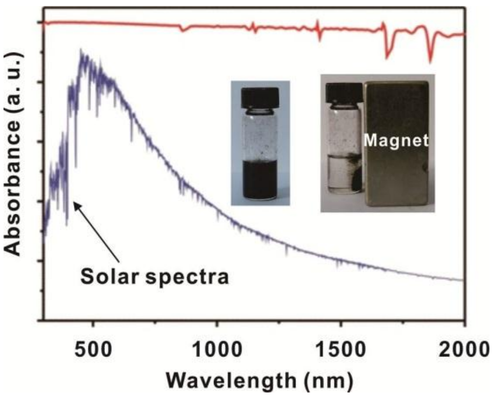

Supplementary Figure 10 | Absorption spectrum of Fe_3O_4@graphene nanoparticles (NPs) dispersed within chloroform. The inset images show that the hybrid NPs in the uniform chloroform dispersion (0.2 mg ml^{-1}) can be attracted to the side by an external magnet.

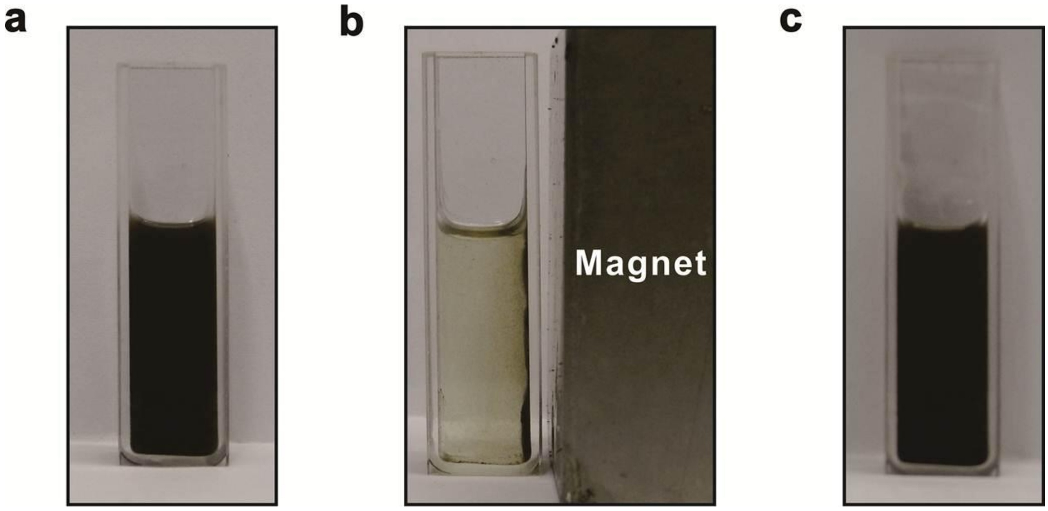

Supplementary Figure 11 | Reversible dispersion of Fe_3O_4@graphene nanoparticles (NPs) within melted paraffin (0.1 wt%) through magnetic manipulation. **a**, A photograph showing homogenous dispersion state of the hybrid NPs in the paraffin. **b**, A photograph showing the hybrid NPs being fully attracted to the side wall with an external magnet. **c**, A photograph of recovered uniform dispersion of the hybrid NPs obtained by rotating the magnet around the sample holder.

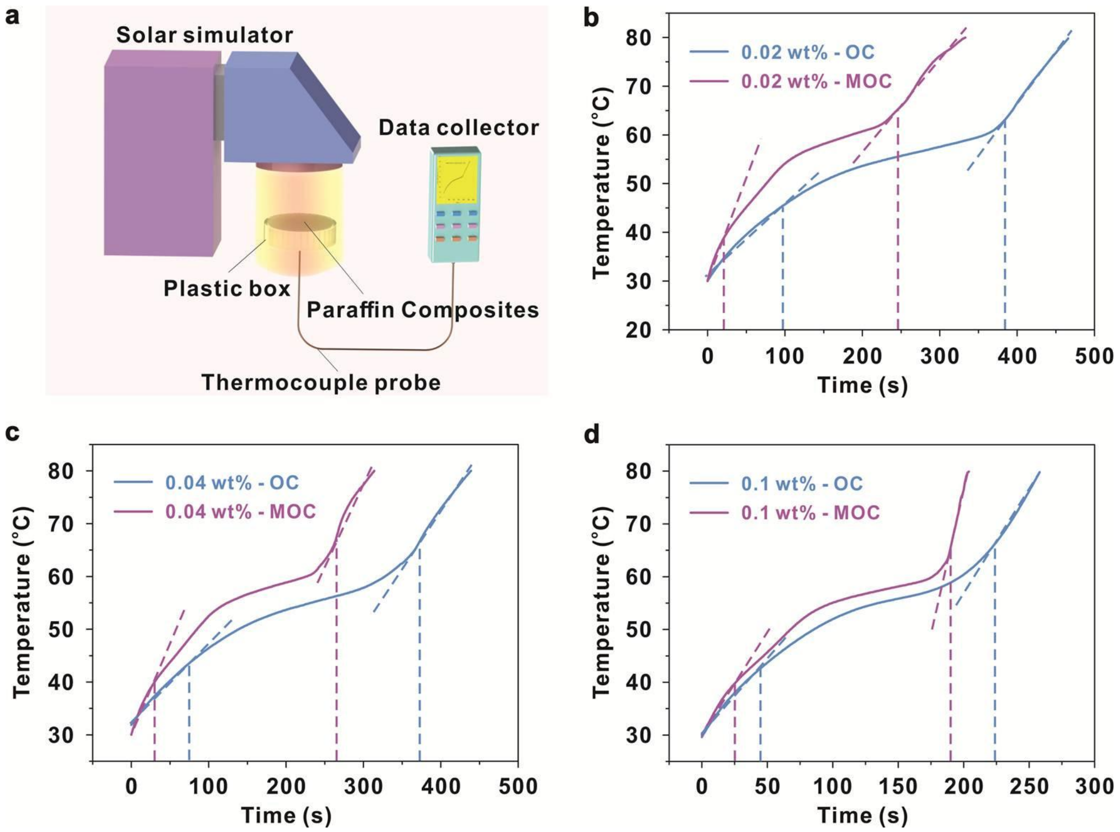

Supplementary Figure 12 | Evaluation of solar-thermal conversion efficiency with a tangential method. a, Scheme of experimental setup. **b, c, d,** Temperature rising curves of paraffin composites with 0.02 wt%, 0.04 wt% and 0.1 wt% of Fe_3O_4@graphene particles during solar radiation under optical charging (OC) and magnetically-enhanced optical charging (MOC) mode.

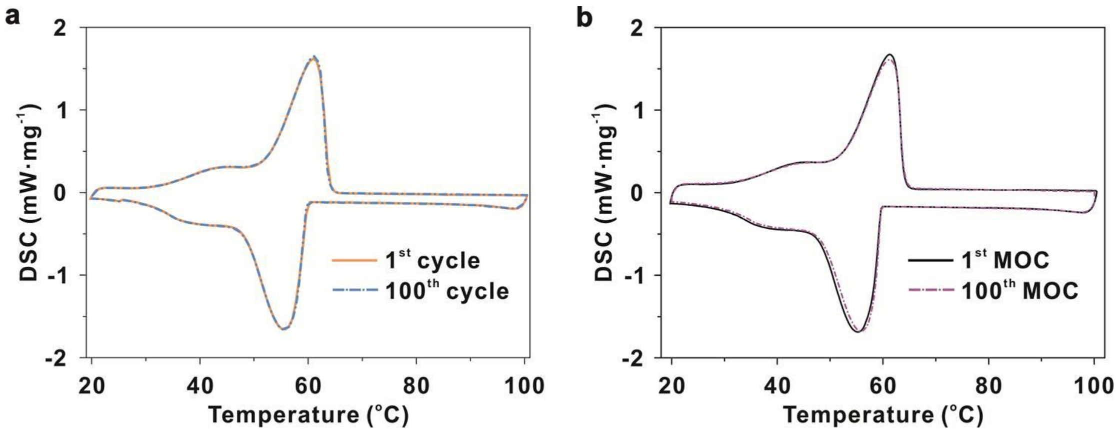

Supplementary Figure 13 | Thermal and repeated optical charging cycling stability. a, Differential scanning calorimetry (DSC) curves of paraffin-Fe_3O_4@graphene composites (0.1 wt%) after repeated heating/cooling for 100 cycles. **b,** DSC curves of paraffin-Fe_3O_4@graphene composites (0.1 wt%) after repeated charging/cooling through magnetically-enhanced optical charging (MOC) process for 100 cycles.

Supplementary Note 1: Preparation of paraffin-Fe_3O_4@graphene composite

The Fe_3O_4@graphene hybrid nanoparticles (NPs) were prepared by referring to the procedure reported by Tuan *et al*[1] with modifications. Graphene oxide (GO) nanosheets were synthesized by a modified Hummers method and used as the starting material to prepare graphene. In a typical synthesis of GO nanosheets, graphite (0.5 g, Alladin Reagent, Shanghai, China), sodium nitrate (0.5 g, Alladin Reagent, Shanghai, China), concentrated sulfuric acid (95%, 40 ml, Alladin Reagent, Shanghai, China), and 3 g of potassium permanganate (Alladin Reagent, Shanghai, China) were sequentially added to a 250 ml round bottom flask that was cooled to 0 ^{o}C. The flask was then transferred to a water bath (40 ^{o}C) and the mixture was stirred for 1 h before being diluted with 130 ml DI water. In the final step, 3 ml of hydrogen peroxide (30% in water, Alladin Reagent, Shanghai, China) was added dropwise into the mixture. The solution was centrifuged and washed until reaching a neutral pH. The precipitated GO nanosheets were finally dried in an oven at 70 ^{o}C.

To synthesize the hybrid NPs, 20 mg of GO was added to the mixture of oleylamine (5 ml) and toluene (15 ml), and sonicated for 40 min. The dispersion was then centrifuged at 8000 rpm for 15 min to separate the functionalized GO from the solvent. The sediment was dried and redispersed in 20 ml dibenzyl ether (Alladin Reagent, Shanghai, China) followed by the addition of 42.4 mg iron acetylacetonate (Alladin Reagent, Shanghai, China). Under the purging of N_2 gas, the whole solution was heated with under stirring at a heating rate of 10 ^{o}C min^{-1} until reaching the boiling point of dibenzyl ether and boiled for 2 h. After cooling down to room temperature, the hybrid NPs were recovered by repeated centrifugation and washing with mixed toluene and ethanol (1:1, volume ratio) and were finally dispersed in chloroform. Paraffin-Fe_3O_4@graphene composites were prepared by mixing chloroform dispersion of Fe_3O_4@graphene NPs with melted liquid-phase paraffin followed by evaporation removal of the chloroform solvent and the solid composites were obtained through natural cooling to room temperature.

Supplementary Note 2: Theoretical modeling of charging process

Simulation of thermal charging (TC) process

An analytical method is applied to investigate the thermal charging process of the thermal energy storage system. The following assumptions have been made: (1) the system is considered as one-dimensional and semi-infinite; (2) constant heat flux boundary condition (Neumann boundary condition) is applied to the left boundary; (3) heat losses due to conduction, radiation, convection, and evaporation are neglected; (4) the free convection of liquid state PCMs is neglected; (5) the physical properties of the PCMs listed in Supplementary Table 1 are isotropic and independent of the temperature.

The formulation of the one-dimensional heat conduction problem is provided as below. The governing energy equation in solid and liquid region can be described by the standard heat diffusion equations:

$$\frac{\partial^2 T_l(x,t)}{\partial x^2} = \frac{1}{\alpha_l}\frac{\partial T_l(x,t)}{\partial t} \text{ in } 0 \le x \le s(t) \quad (1)$$

$$\frac{\partial^2 T_s(x,t)}{\partial x^2} = \frac{1}{\alpha_s}\frac{\partial T_s(x,t)}{\partial t} \text{ in } s(t) \le x \le \infty \quad (2)$$

where T_i, k_i and $\alpha_i = \frac{k_i}{\rho_i c_i}$ are temperature, thermal conductivity, and thermal diffusivity of the i th phase, respectively ($i = l$ (liquid) or s (solid)). ρ_i and c_i are the density and specific heat of the i th phase and $s(t)$ is the position of solid-liquid interface at time t. The boundary conditions are

$$-k_l \frac{\partial T_l(x,t)}{\partial x} = q_0 \quad \text{at } x = 0 \quad (3)$$

$$T_s(x,t) = T_0 \quad \text{at } x \to \infty \quad (4)$$

where q_0 is the heat flux at the left boundary and T_0 is the initial temperature.

The interface conditions are:

$$T_l(x,t) = T_s(x,t) = T_m \quad \text{at } x = s(t) \quad (5)$$

$$\rho L \frac{ds(t)}{dt} = k_s \frac{\partial T_s(x,t)}{\partial x} - k_l \frac{\partial T_l(x,t)}{\partial x} \quad \text{at } x = s(t) \quad (6)$$

where T_m is the melting temperature and L is the latent heat.

For the initial condition, we assume the PCM has a uniform temperature and specify

$$T_s(x,0) = T_0 \quad (7)$$

From the above equations, we can obtain the analytical solution of the temperatures for solid and liquid phases, respectively:

$$T_l(x,t) = T_m + \frac{(\pi\alpha_l)^{1/2} q'}{k_l}[\text{erf}(\lambda) - \text{erf}(\frac{x}{2(\alpha_l t)^{1/2}})] \quad (8)$$

$$T_s(x,t) = T_0 + (T_m - T_0)\frac{\text{erfc}[x/2(\alpha_s t)^{1/2}]}{\text{erfc}[\lambda(\alpha_l/\alpha_s)^{1/2}]} \quad (9)$$

where erf( ) and erfc( ) are the error function and complementary error function respectively, $q' = \sqrt{t} q_0$ and $\lambda = \frac{s(t)}{2(\alpha_l t)^{1/2}}$. In order to satisfy the interface condition Supplementary Equation 6, λ must satisfy the following transcendental equation

$$e^{-\lambda^2}q' - \frac{e^{-\lambda^2(\alpha_l/\alpha_s)}}{\mathrm{erfc}[\lambda(\alpha_l/\alpha_s)^{1/2}]}\frac{(T_m - T_0)k_s}{(\pi\alpha_s)^{1/2}} = \rho L\lambda(\alpha_l)^{1/2} \tag{10}$$

To model the thermal diffusion charging process in our experiment, the initial temperature was set to be 20 ^{o}C and the heat flux is 4 W cm^{-2} according to the experimental laser power input. The thermophysical properties of paraffin wax were experimentally measured and used as the input parameters for modeling. Specifically, the densities of paraffin at solid and liquid phase were determined by a density meter. Heat capacity (c), latent heat (L), onset melting temperature (T_m) were measured by Differential scanning calorimetry (DSC, Netzsch 204 F1). Thermal conductivities (k) at solid state (k_s) and liquid state (k_l) were measured by a transient hot bridge analyzer (Linseis THB-1, Linesis Inc., Germany) and a hot wire method with a home-built setup[2], respectively. All the measured data are listed in Supplementary Table 1.

Simulation of optical charging (OC) process

Optical charging process is more complicated compared to the thermal charging process, because the heat source (i.e., absorbed light) is moving along with the interface. It is difficult to solve this problem analytically or numerically. To theoretically investigate the optical charging process, we developed a 'step-by-step' model based on control volume and energy conservation. The following assumptions are made: (1) the system is considered as one-dimensional and semi-infinite; (2) the energy distribution of the laser beam in the liquid region follows the Beer–Lambert law; (3) the thermal conduction in the PCMs can be neglected; (4) heat losses due to conduction, radiation, convection, and evaporation are not considered; (5) the physical properties of the PCMs listed in Supplementary Table 1 are isotropic and independent of the temperature. The assumption that thermal conduction is negligible is based on the fact that the conduction heat flux is much smaller than the heat generation induced by laser illumination.

Due to the optical absorption of NPs in the liquid region, light will attenuate in liquid portion of the melted composite. The remaining energy is absorbed by the solid PCMs to induce phase change. In our model, the simulation domain is divided into small slices (control volumes) with a thickness of Δx. During the optical charging process, each slice melts after a certain illumination duration, and then the light starts to melt the next slice. Our 'step-by-step' model is explained as follows.

First, light attenuates in the liquid region following the Beer–Lambert law:

$$I = I_0 \exp(-\alpha x) \tag{11}$$

where α is the absorptivity of liquid PCM and I_0 is the power density of the laser beam. Because light energy is partly absorbed by the liquid before reaching the solid-liquid interface, the heat flux to melt the solid region becomes smaller at larger x. For the first slice

in the solid region (the n-th slice), we assume the laser beam induces a uniform heating within this control volume. By neglecting heat conduction, the energy conversion can be written as

$$I_0 \exp[-\alpha(n-1)\Delta x]t_n = \rho\Delta x[L + c(T_m - T_0)] \tag{12}$$

The left side of Supplementary Equation 12 represents the energy absorbed in the control volume. The right side is the energy needed to melt the solid in the control volume, including the sensible heat and latent heat.

After melting of the n-th slice, the position of solid-liquid interface moves to position $x_n = n\Delta x$, and the corresponding illumination time $t(x_n)$ is the sum of total time to melt the first n slices, given by

$$t(x_n) = \sum_{i=1}^{n} t_i = \frac{\rho x_n[L + c(T_m - T_0)]}{nI_0} \cdot \frac{1 - \exp(x_n\alpha)}{1 - \exp(\alpha x_n / n)} \tag{13}$$

For the liquid region, the heating is induced by the NPs that absorb part of the laser energy. The energy conversion for the i-th control volume in the liquid region is determined by

$$I_0\{\exp[-\alpha(i-1)\Delta x] - \exp(-\alpha i\Delta x)\}(\sum_{j=1}^{n} t_j - \sum_{j=1}^{i} t_j) = \rho c\Delta x(T_i - T_m) \tag{14}$$

By solving Supplementary Equation 14, we can obtain the temperature distribution in liquid region

$$T_i = \frac{L + c(T_m - T_0)}{c}\{\exp[(n-i)\alpha\Delta x] - 1\} + T_m \tag{15}$$

The temperature in solid region is assumed to be the initial temperature of 20 °C .

To solve Supplementary Equation 12, we actually replace the laser illumination with a uniform heat flux in the region Δx . To determine the appropriate step size we tried different Δx . The results show that when Δx approaches 0.01 cm, the simulated propagation curves of charging interface do not change much (Supplementary Fig. 3a). Based on Supplementary Fig. 3a, a step size of 0.01 cm was used in our model to simulate the charging process with appropriate amount of computation effort. The initial temperature is assumed to be 20 °C and the power density of incident laser beam is 4 W cm^{-2}. The absorption coefficient of the liquid-phase composite (α) was determined by measuring the power attenuation of laser beam after passing through the melted composites with a power meter based on the Beer–Lambert law.

Supplementary Note 3: Heat loss analysis

Heat loss from the PCM system

In the experimental system, as the light transmitted through the front surface of the quartz container, there will be optical losses through reflection at the surface of the quartz (approximately 8.5%, Supplementary Fig. 6a). For the TC mode, the reflection at the surface

of the black aluminum absorber (approximately 4%, Supplementary Fig. 6a) further reduced the light intensity reaching the PCM system.

After the light was absorbed either by the black aluminum absorber in TC mode or by the PCM in the OC and MOC mode, the light energy will be converted into thermal energy (Q_{input}). Part of the Q_{input} is used to charge the PCM, which is Q_{storage}. The difference between Q_{input} and Q_{storage} is the heat loss (Q_{loss}). As shown in Supplementary Fig. 6b, the Q_{loss} consists of the following components:

(1) The heat loss from the top surface of the PCM---$Q_{\mathrm{loss}}^{\mathrm{PCM\_top}}$

(2) The heat loss from the PCM to the quartz container, and such heat loss has the following components:

  (a) The heat loss that is used to heat up the container, which is $Q_{\mathrm{loss}}^{\mathrm{container\_sensible}}$. The heat conducted from PCM increases the temperature of the quartz container. Quartz has a relatively larger thermal conductivity (approximately 1.3 W m^{-1} K^{-1}) than the PCM (approximately 0.2 W m^{-1} K^{-1}). Heat thus can spread into much larger area in the walls of the quartz container than in the PCM. Such spreading increases the amount of quartz that is heated up during the charging process.

  (b) The heat loss from the front of the container, which is $Q_{\mathrm{loss}}^{\mathrm{container\_front}}$. Considering the thickness (1.5 mm) and the height (5.5 mm) of the quartz wall, the surface area for this heat loss ($Q_{\mathrm{loss}}^{\mathrm{container\_front}}$) is actually much larger than the surface area of the front end of the PCM (4 mm × 4 mm). As shown in Supplementary Fig. 6b, the height of the quartz wall (5.5 mm) is designed to be larger than the height of PCM (4 mm) to prevent the spill of the PCM during the melting process, especially at high temperatures. The $Q_{\mathrm{loss}}^{\mathrm{container\_front}}$ includes the convection and radiation heat loss from the outer front surface and also the convection and radiation heat loss from the inner front surface, both of which are exposed to air during the charging process.

  (c) Heat losses from other parts of the quartz container, which is $Q_{\mathrm{loss}}^{\mathrm{container\_other}}$, including the heat loss from the two sides, bottom, and backside of the container. Considering the relatively low temperature shown at the backside of the container, we only considered the heat losses from the two sides and the bottom of the container for this portion of heat loss. The heat loss at the two sides includes the heat loss from both the outer and inner surfaces of the quartz container. The inner surfaces are exposed to air so there are convection and radiation heat losses. The two side surfaces at the outer and the bottom surface of the container are in direct contact with the thermal insulation, so the convection and radiation heat loss is suppressed, but there is still heat flow from the heated quartz surfaces to the insulation foam. We did observe the heating up of the insulation foam during the experiment. With the large surface area of the side and bottom surfaces, and also the spreading of the heat in the quartz wall due to the relatively large thermal conductivity of the quartz, which further increases the surface area for the heat loss,

the heat loss from the two sides and the bottom of the container is relatively substantial, so this part of heat loss is also considered in our analysis.

Estimation of the heat loss

Based on the above analysis of the heat loss, in this section we tried to use the following equations to estimate each part of the heat losses:

(1) $Q_{\text{loss}}^{\text{PCM\_top}}$

Heat loss from the top surface of the charged PCM could be calculated by:

$$Q_{\text{loss}}^{\text{PCM\_top}} = \int_0^t h_{\text{loss}}^{\text{PCM\_top}} A_{\text{top}}\left(T_{\text{ave\_top}} - T_\infty\right) dt' = h_{\text{loss}}^{\text{PCM\_top}} \int_0^t A_{\text{top}}\left(T_{\text{ave\_top}} - T_\infty\right) dt' \quad (16)$$

where A_{top} is the top surface area of the PCM, $T_{\text{ave\_top}}$ is the average top surface temperature of the charged PCM, and T_∞ is the ambient temperature. Here we used an average temperature ($T_{\text{ave\_top}}$) and an average heat transfer coefficient ($h_{\text{loss}}^{\text{PCM\_top}}$) to calculate this portion of the heat loss.

(2) $Q_{\text{loss}}^{\text{container\_sensible}}$

The sensible heat gained by quartz container during the heating up of the container $\left(Q_{\text{loss}}^{\text{container\_sensible}}\right)$ could be calculated by:

$$Q_{\text{loss}}^{\text{container\_sensible}} = c_q \rho_q d A_{\text{f}}\left(T_{\text{ave\_front}} - T_0\right) + 2 c_q \rho_q d A_s\left(T_{\text{ave\_side}} - T_0\right) + c_q \rho_q d A_{\text{bottom}}\left(T_{\text{ave\_bottom}} - T_0\right) \quad (17)$$

where d is the thickness of the quartz wall (1.5 mm), ρ_q is the density of quartz (2.203 kg m^{-3}), and c_q is the specific heat of quartz (0.7 J g^{-1} K^{-1}). A_{f} (= $A_{\text{f\_outer}}$ in Supplementary Fig. 6b), A_s (= $A_{\text{s\_outer}}$ in Supplementary Fig. 6b) and A_{bottom} are the area of front, side and bottom surfaces of the container, respectively. $T_{\text{ave\_front}}$, $T_{\text{ave\_side}}$ and $T_{\text{ave\_bottom}}$ are the average temperature of front, side and bottom surfaces of the container, respectively. T_0 is the initial temperature of the container. In the second term, the factor of 2 for A_s is to account for two side walls of the container. Here we use the average temperature for each surface to estimate the sensible heat in the quartz walls.

(3) $Q_{\text{loss}}^{\text{container\_front}}$

Heat loss from the quartz container front surface could be estimated by:

$$Q_{\text{loss}}^{\text{container\_front}} = \int_0^t h_{\text{loss}}^{\text{quartz\_front}} \left[\left(A_{\text{f\_outer}} + A_{\text{f\_inner}}\right)\left(T_{\text{ave\_front}} - T_\infty\right)\right] dt'$$

$$= h_{\text{loss}}^{\text{quartz\_front}} \int_0^t \left(A_{\text{f\_outer}} + A_{\text{f\_inner}}\right)\left(T_{\text{ave\_front}} - T_\infty\right) dt' \quad (18)$$

Again, we also use the average temperatures and an average $h_{\text{loss}}^{\text{quartz\_front}}$ for the estimation.

(4) $Q_{\text{loss}}^{\text{container\_other}}$

Besides the front surface, heat could also be dissipated through other parts of the container that were also heated up. The heat losses from these parts are a little complex, with the inner surfaces exposed to air, while the outer surfaces are insulated. To simplify the estimation, we also used an average heat transfer coefficient ($h_{\text{loss}}^{\text{quartz\_other}}$) to estimate this portion of heat loss from the side walls and the bottom of the quartz container.

$$Q_{\text{loss}}^{\text{container\_other}} = \int_0^t h_{\text{loss}}^{\text{quartz\_other}} [2(A_{\text{s\_outer}} + A_{\text{s\_inner}})(T_{\text{ave\_side}} - T_\infty) + A_{\text{b}}(T_{\text{ave\_bottom}} - T_\infty)]\, dt'$$

$$= h_{\text{loss}}^{\text{quartz\_other}} \int_0^t [2(A_{\text{s\_outer}} + A_{\text{s\_inner}})(T_{\text{ave\_side}} - T_\infty) + A_{\text{b}}(T_{\text{ave\_bottom}} - T_\infty)]\, dt' \quad (19)$$

We used an overall average heat loss coefficient ($h_{\text{loss-ave}}$) to replace all the heat loss coefficients in the above equations. Following are the new equations with this average heat transfer coefficient ($h_{\text{loss\_ave}}$):

$$Q_{\text{loss}}^{\text{PCM\_top}} = h_{\text{loss\_ave}} \int_0^t A_{\text{top}}(\mathrm{T}_{\text{ave\_top}} - T_\infty)\, dt' \quad (20)$$

$$Q_{\text{loss}}^{\text{container\_front}} = h_{\text{loss\_ave}} \int_0^t (A_{\text{f\_outer}} + A_{\text{f\_inner}})(T_{\text{ave\_front}} - T_\infty)\, dt' \quad (21)$$

$$Q_{\text{loss}}^{\text{container\_other}} = h_{\text{loss\_ave}} \int_0^t [2(A_{\text{s\_outer}} + A_{\text{s\_inner}})(T_{\text{ave\_side}} - T_\infty) + A_{\text{b}}(T_{\text{ave\_bottom}} - T_\infty)]\, dt' \quad (22)$$

The overall energy balance equation can thus be written as:

$$Q_{\text{input}} - Q_{\text{storage}} = Q_{\text{loss}} \quad (23)$$

$$Q_{\text{loss}} = Q_{\text{loss}}^{\text{PCM\_top}} + Q_{\text{loss}}^{\text{container\_sensible}} + Q_{\text{loss}}^{\text{container\_front}} + Q_{\text{loss}}^{\text{container\_other}} = Q_{\text{loss}}^{\text{container\_sensible}} + h_{\text{loss\_ave}} \int_0^t [(A_{\text{f\_outer}} + A_{\text{f\_inner}})(T_{\text{ave\_front}} - T_\infty) + 2(A_{\text{s\_outer}} + A_{\text{s\_inner}})(T_{\text{ave\_side}} - T_\infty) + A_{\text{b}}(T_{\text{ave\_bottom}} - T_\infty) + A_{\text{top}}(\mathrm{T}_{\text{ave\_top}} - T_\infty)]\, dt' \quad (24)$$

With the known light intensity, and the optical losses due to the reflection at the quartz surface and the surface of the black aluminum absorber, Q_{input} can thus be calculated. Q_{storage} can be estimated through the similar analysis as shown in Supplementary Fig. 7, and thus Q_{loss} can be estimated through Supplementary Equation 23. From Supplementary Equation 24, if we can estimate $Q_{\text{loss}}^{\text{container\_sensible}}$ and obtain the average temperature of all the surfaces, we can then estimate the $h_{\text{loss\_ave}}$.

To calculate $Q_{\text{loss}}^{\text{container\_sensible}}$, we will need to have the average temperature for all the surfaces of the quartz container, which is challenging to obtain for the sample with the thermal insulation. We first tried to estimate $Q_{\text{loss}}^{\text{container\_sensible}}$ without thermal insulation since we could use IR camera to map out the temperature profile of all the surfaces of the quartz container, and then obtain the average temperatures for all the surfaces of the quartz

container. Supplementary Fig. 6c shows the average temperature of the front and the side surfaces of the quartz container without the thermal insulation. With the heat spreading to the area of $A_{\mathrm{f\_outer}}$(the outside surface area of the quartz container), which is larger than the heat receiving surface area (front end of the PCM), the average temperature of $A_{\mathrm{f\_outer}}$ is thus lower than the maximum top surface temperature of the PCM.

Supplementary Fig. 6c shows that the average temperature of the front surface of the quartz container for the TC mode is much higher than those for the OC mode and MOC mode. Such temperature difference not only leads to the smallest convection and radiation heat loss at the front ($Q_{\mathrm{loss}}^{\mathrm{container\_front}}$) for the MOC mode, but also the smallest heat loss to heat up the quartz container ($Q_{\mathrm{loss}}^{\mathrm{container\_sensible}}$) for the MOC mode. We observed the similar trend in the side surfaces of the quartz container (Supplementary Fig. 6d), which also leads to the smallest heat loss for the MOC mode.

With the obtained temperature profiles through IR imaging, the average temperature for all quartz surfaces can thus be calculated. We used the temperature profile in the side surfaces that was close to the bottom of the container to estimate the average temperature of the bottom of the container. The $Q_{\mathrm{loss}}^{\mathrm{container\_sensible}}$ can thus be calculated using Supplementary Equation 17 and the average heat loss coefficient ($h_{\mathrm{loss\_ave}}$) can then be calculated using Supplementary Equation 24. The $h_{\mathrm{loss\_ave}}$ was estimated to be approximately 10.0 W m^{-2} K^{-1} for TC mode, approximately 9.2 W m^{-2} K^{-1} for OC mode, and approximately 9.1 W m^{-2} K^{-1} for MOC mode, respectively. With the estimated $h_{\mathrm{loss\_ave}}$, we also calculated the heat loss contributions from all the quartz surfaces and the PCM using Supplementary Equation 20-22, and plotted them together with $Q_{\mathrm{loss}}^{\mathrm{container\_sensible}}$ in Supplementary Fig. 8. In Supplementary Fig. 8, $Q_{\mathrm{loss}}^{\mathrm{container\_other}}$ in TC mode was used as the benchmark. As shown in Supplementary Fig. 8, for MOC mode, the heat loss that heats up the quartz container is the smallest among all three charging modes. The same is true for the heat loss from both the front surfaces and other surfaces of the quartz container. Supplementary Fig. 8 represents the case without the thermal insulation, but it does show that besides the heat loss at the front surface of the quartz container, there are other key heat loss mechanisms ($Q_{\mathrm{loss}}^{\mathrm{container_sensible}}$and $Q_{\mathrm{loss}}^{\mathrm{container\_other}}$) that contributes to the smaller heat loss for the MOC mode. With the thermal insulation, the quartz container will be heated up more so $Q_{\mathrm{loss}}^{\mathrm{container\_sensible}}$ will be larger than in the case without the thermal insulation. With the thermal insulation, the quartz surfaces that are heated will also heat up the polystyrene foam that is used as the thermal insulation. In the experiment, we embedded a thermocouple into the insulation foam near the front charging face (close to the same height as the center of the PCM and approximately 1.5 mm away from the quartz container outer wall) to measure the temperature of the foam during the charging process. After charging for 270 s, the insulation foam was heated up to 60 ^{o}C, 45°C and 40 ^{o}C for TC, OC and MOC mode, respectively. Considering the relatively large surface areas of the quartz container that were insulated, and also the heat spreading within the quartz wall that further increased the

heated surface area of the quartz, the heat loss from the quartz surface to the insulation foam would be quite substantial as well for the system with the thermal insulation.

Simulation of temperature distribution with heat losses

For OC and MOC mode, we tried to calculate the temperature distribution by taking into consideration of the heat losses. Based on the heat loss analysis discussed above, we added two heat loss terms in the Supplementary Equation 14 shown below: one is related to the heat loss that heats up the quartz container, and the other is the heat loss that accounts for all other components of the heat losses.

$$\{I_0[\exp[-\alpha(i-1)\Delta x]-\exp[-\alpha i\Delta x]]-h_{\text{loss\_ave}}p\Delta x(T_i-T_\infty)/A_c\}\left(\sum_{j=1}^{n}t_j-\sum_{j=1}^{i}t_j\right)-\rho_q c_q dl\Delta x(T_i-T_m)/A_c=\rho_l c_l\Delta x(T_i-T_m) \quad (25)$$

where p is the perimeter of the cuvette, A_c is the PCM section area (0.004 × 0.004 m^2), T_∞ is the ambient temperature, T_m is the melting temperature of PCM, d is the thickness of the quartz container (1.5 mm), ρ_q is the density of quartz (2.203 kg m^{-3}), c_q is the specific heat (0.7 J g^{-1} K^{-1}), l (3 × 0.004 m) is the length of the quartz wall that surrounds the PCM slice. In this equation, we assume that the quartz walls are in thermal equilibrium with the PCM and the temperature of the quartz walls is the same as the temperature of the PCM.

In Supplementary Equation 25, we first used the estimated average heat loss coefficient ($h_{\text{loss\_ave}}$) for the OC and MOC mode to calculate the temperature distribution of the PCM for the OC and MOC mode. Such average $h_{\text{loss\_ave}}$ was estimated without the thermal insulation, so heat loss occurred at all the surfaces of the quartz container and also the top of PCM. To estimate the $h_{\text{loss\_ave}}$ with the thermal insulation, we consider an extreme case in which the thermal insulation is an ideal thermal insulation. For such ideal thermal insulation, it does not absorb any heat from the heated quartz walls, and it prevents all the heat losses from the heated quartz walls that are in contact with such thermal insulation. In this case, besides $Q_{\text{loss}}^{\text{container\_sensible}}$, the rest of Q_{loss} can only be dissipated through the exposed surface of the quartz container. Using such insulation, the estimated $h_{\text{loss\_ave}}$ represents a high boundary of $h_{\text{loss\_ave}}$. By using Supplementary Equation 24, and using only the surface areas exposed to air, we estimated such high boundary of $h_{\text{loss\_ave}}$ to be 20.5 m^{-2} K^{-1} for TC mode, 18.2 m^{-2} K^{-1} for OC mode, and 17.8 m^{-2} K^{-1} for MOC mode. We used these $h_{\text{loss\_ave}}$ for the OC and MOC mode to calculate the temperature distribution profiles of the PCM in the OC and MOC mode as well.

In the simulation of the temperature distribution for TC mode using Fluent, we also considered the heat loss that heats up the quartz container and the heat loss dissipates at the other parts of the system. In Fluent, these heat losses were considered in the boundary conditions. The heat loss that heats up the quartz container was calculated using Supplementary Equation 17, and such heat loss ($Q_{\text{loss}}^{\text{container\_sensible}}$) was accounted in the input light energy (Q_{input}) in the simulation of the temperature distribution for the TC mode. The other parts of the heat loss were considered to be dissipated at the surfaces exposed to

air during the simulation using Fluent. For those other parts of the heat loss, an average heat loss coefficient $h_{\text{loss\_av}}$ of 10.0 m^{-2} K^{-1} at the lower boundary and 20.5 m^{-2} K^{-1} at the higher boundary was used in the calculation of the temperature distribution of the PCM in the TC mode.

Supplementary Fig. 9 shows the calculated temperature distributions using both the low and high boundary $h_{\text{loss\_ave}}$ for all three charging modes. The inset plots represent the experimentally measured temperature distributions. By considering the heat losses, the calculated temperature distributions are getting closer to the experimental measurement. In general, the temperature at the starting position calculated using the higher boundary of the estimated $h_{\text{loss\_ave}}$ is smaller than the temperature measured in the experiment, while such temperature calculated using the lower boundary of the estimated $h_{\text{loss\_ave}}$ is larger than the temperature measured in the experiment, which implies that the $h_{\text{loss\_ave}}$ for the PCM system might be between those two estimated values.

Supplementary Note 4: Calculation of stored solar-thermal energy

Based on the final temperature distribution profiles, the amount of stored solar-thermal energy including the sensible heat stored within solid PCM, the sensible heat stored within liquid PCM, and the latent heat were calculated. The stored sensible heat was calculated through integrating the temperature profile along the charging distance by using $H_{\text{sensible}} = \int_{x_1}^{x_2} c_{\text{p}}(T - T_0)\rho A_{\text{c}} d_{\text{x}}$, where ρ is the density of PCM (liquid: 910 kg m^{-3}, solid: 976 kg m^{-3}), c_{p} is the heat capacity of PCM (liquid: 2.8 J g^{-1} K^{-1}, solid: 2.4 J g^{-1} K^{-1}), T_0 is the initial temperature of PCM, A_{c} is the cross-sectional area of the PCM sample, and x is the sample length along the charging direction. In Fig. 4b, for the sensible heat stored within liquid PCM under OC mode, x_1 and x_2 are 0 and 1.3 cm, respectively. For the sensible heat stored within solid PCM, x_1 and x_2 are 1.3 and 2 cm, respectively. The latent heat stored was estimated by using the mass in liquid phase and the latent heat of fusion: $H_{\text{latent}} = M_{\text{liquid}} \times H_{\text{fusion}} = \rho \times A \times (x_2 - x_1) \times H_{\text{fusion}}$, where $(x_2 - x_1)$ stands for the melted length of the sample. The heat of fusion (H_{fusion}) for PCM composites with 0.02 wt% of Fe_3O_4@graphene hybrid NPs was 174 J g^{-1} based on the DSC measurement. The same approach was used to calculate the stored solar-thermal energy under TC and MOC mode. The relative amount of stored energy was compared among different charging modes.

Supplementary Note 5: Phase-change solar-thermal energy storage efficiency calculation

As shown by Supplementary Fig. 12a, thin composite samples with thicknesses of approximately 3 mm were first placed in a transparent plastic petri dish (with a diameter of 4 cm) that was thermally insulated with polystyrene foam and illuminated by simulated solar light under a power density of 0.3 W cm^{-2}. The temperature evolution was monitored

by the thermocouple, which was inserted into the PCM and recorded by a data acquisition system (Agilent 34972A, Agilent Technologies, USA). Thermal energy storage efficiency during the phase change process was calculated based on the ratio of the stored thermal energy in paraffin wax to the optical illumination energy received by the composite material over the phase change process. The efficiency can be described by the following formula:

$$\eta = \frac{m\,\Delta H}{Pt} \tag{26}$$

where m is the weight of the charged sample, ΔH is heat of fusion of the optically charged composite samples that is determined by DSC measurement; P is the solar power that can be calculated by multiplying solar power density with the area of the sample; t is the charging time during the phase change process. The phase-change period (t) was determined by the tangential method through the analysis of the heating curve[3-9] and was defined as the time difference between the starting point and the end point of the phase change process. Under MOC mode, the magnet with a magnetic field strength of approximately 0.3 T was placed along the side wall of the petri dish and the sample was illuminated with the same solar light. Changing from OC to MOC charging mode, the energy storage efficiency during the phase change process was improved from 57.2% to 75.0%, 67.5% to 83.3%, and 84.2% to 92.4% for paraffin composites loaded with 0.02 wt%, 0.04 wt% and 0.1 wt% Fe_3O_4@graphene, respectively. The use of thin samples, which are needed to ensure the uniform temperature distribution within the samples, in this efficiency evaluation approach could not show the full benefits of MOC process over the OC process since there is less a need to remove the photon absorber from the melted PCM for these thin samples during the charging process.

Supplementary Table 1 Parameters for optical and thermal charging simulation

Parameter	Paraffin	
	Solid	Liquid
ρ (kg m^{-3})	976	910
c (J kg^{-1} K^{-1})	2400	2800
k (W m^{-1} K^{-1})	0.24	0.23
α_{OC} (cm^{-1})	2.66	1.1
α_{MOC} (cm^{-1})	2.66	0.7
L (J kg^{-1})	200000	
T_m (^{o}C)	50	
P (W m^{-2})	40000	

Supplementary references

1. Pang, D. W. P., Yuan, F. W., Chang, Y. C., Li, G. A., & Tuan, H. Y. Generalized syntheses of nanocrystal–graphene hybrids in high-boiling-point organic solvents. *Nanoscale* **4,** 4562-4570 (2012).
2. Zhang, P., Ma, Z. W., Shi, X., & Xiao, X. Thermal conductivity measurements of a phase change material slurry under the influence of phase change. *Int. J. Therm. Sci.* **78,** 56-64 (2014).
3. Chen, L. J. *et al.* Electro- and photodriven phase change composites based on wax-infiltrated carbon nanotube sponges. *ACS Nano* **6,** 10884-10892 (2012).
4. Wang, W. *et al*. Fe_3O_4-functionalized graphene nanosheet embedded phase change material composites: efficient magnetic-and sunlight-driven energy conversion and storage. *J. Mater. Chem. A* **5**, 958-968 (2017).
5. Wang, Y., Tang, B., & Zhang, S. Single-walled carbon nanotube/phase change material composites: sunlight-driven, reversible, form-stable phase transitions for solar thermal energy storage. *Adv. Funct. Mater.* **23,** 4354-4360 (2013).
6. Li, Y., Samad, Y. A., Polychronopoulou, K., Alhassan, S. M., & Liao, K. From biomass to high performance solar-thermal and electric-thermal energy conversion and storage materials. *J. Mater. Chem. A* **2,** 7759-7765 (2014).
7. Zhang, L., Li, R., Tang, B., & Wang, P. Solar-thermal conversion and thermal energy storage of graphene foam-based composites. *Nanoscale* **8,** 14600-14607 (2016).
8. Wang, Y., Tang, B., & Zhang, S. Novel organic solar thermal energy storage materials: efficient visible light-driven reversible solid–liquid phase transition. *J Mater. Chem*. **22,** 18145-18150 (2012).
9. Wang, Y., Tang, B., & Zhang, S. Organic, cross-linking, and shape-stabilized solar thermal energy storage materials: a reversible phase transition driven by broadband visible light. *Appl. Energy* **113,** 59-66 (2014).
